# Is Adherence Theory for Patients With Chronic Disease Robust?: A Critical Evaluation in Patients After Percutaneous Coronary Intervention

**DOI:** 10.1097/JCN.0000000000001232

**Published:** 2025-06-20

**Authors:** Outi Kähkönen, Jani Rannanpää

**Affiliations:** Outi Kähkönen, PhD, Senior Researcher, Department of Nursing Science, Faculty of Health Sciences, University of Eastern Finland, Kuopio, Finland.; Jani Rannanpää, MSc, University Teacher and Biostatistician, School of Computing, University of Eastern Finland, Kuopio, Finland.

**Keywords:** adherence to treatment, nursing theory, structural equation modeling

## Abstract

**Background:**

A comprehensive understanding of the strategies that promote adherence is important for achieving optimal health outcomes in patients after percutaneous coronary intervention.

**Objective:**

The aim of this study was to investigate the associations within Adherence Theory for patients with chronic disease after percutaneous coronary intervention during a long-term follow-up.

**Design:**

A descriptive and exploratory study design was used in this study, with a survey conducted at 2 time points over 6 years.

**Methods:**

Structural equation modeling was used to evaluate the model with baseline data from 2013 (n = 416) and follow-up data from 2019 (n = 167). The instruments used included the Adherence of Patients with Chronic Diseases and Social Support of People with Coronary Heart Disease questionnaires.

**Results:**

Adherence Theory for patients with chronic disease showed a robust structure with no significant differences between 2013 and 2019. However, significant gender differences were observed in both years. Motivation was directly associated with adherence in 2013. For men, indirect associations were found between informational support, sense of normality, support from next of kin, and results of care. Adherence for women was not influenced by any identifiable factors in 2019, as observed in 2013. Among men, motivation was directly associated with adherence and indirectly linked to a sense of normality, fear of complications, and support from next of kin.

**Conclusions:**

The results underscore the importance of critically reassessing nursing theories to ensure their relevance. This research-based knowledge can then be used to develop nursing interventions that promote person-centered care.

**What’s New and Important**

The study’s results emphasize the importance of critically reevaluating the nursing theories by enhancing and integrating holistic, person-centered care that account for gender differences.

- Adherence Theory for patients with chronic disease is robust over the long term in evaluating adherence to treatment after percutaneous coronary intervention.
- Significant gender differences exist in factors associated with adherence to treatment. Nursing practice should address these differences and offer targeted, gender-specific interventions.
- Informational support and the social environment are critical factors that influence adherence differently in men and women.

Coronary heart disease (CHD) is a serious challenge to global public health, resulting in financial costs and considerable human suffering. Despite advanced treatment options that reduce the burden of disease, CHD remains prevalent. The treatment of CHD generally involves 3 primary strategies: lifestyle changes, optimal medication management, and revascularization through percutaneous coronary intervention (PCI) or coronary artery bypass grafting. These treatments are complementary rather than mutually exclusive.^1^

Along with optimal medical and lifestyle management, PCI has become the most commonly used revascularization procedure for obstructive CHD in patients with persistent angina and acute coronary syndrome. The benefits of PCI include rapid recovery, improved overall health, and enhanced quality of life. It is critical to evaluate and refine our theoretical understanding of adherence and its determinants to effectively address the needs of patients after PCI.^2^

Nursing is based on both theory and practice. Nursing theories provide a structured framework that defines the core values and beliefs nurses uphold regarding health and patient care, guiding their practice and decision-making.^3^ The study of nursing theories is critical because it provides the basis for evidence-based interventions. These theories provide a framework for clinical nursing practice by offering theoretical evidence that validates specific actions^2^ and providing validated methods for relating observed phenomena to the theory’s underlying knowledge, effectiveness, and generalizability of the theory.^4^ In addition, they justify, strengthen, and promote care that is both comprehensive and person centered. Despite their importance, concerns have been raised about the future of nursing knowledge articulated through theory.^5^

Nursing theories are facing challenges due to rapid changes in society, healthcare, and science. It has been proposed that the accelerated pace of change requires a comprehensive reassessment of the theories that underpin nursing practice. This is to ensure that traditional approaches are not superseded by new developments and that the nursing discipline continues to advance.^6^

Current challenges include defining the discipline’s knowledge structure, establishing a coherent focus, and redefining theory’s role in nursing science to guide future practice development. A theory-based approach to healthcare is imperative for understanding how and under what conditions nursing interventions lead to meaningful change,^2^ while at the same time advancing nursing research by providing deeper insights into the effectiveness of these interventions.^7^ The absence of a theoretical nursing perspective in practice can also lead to substandard care and an increased risk of negative outcomes.^8^

Given the importance of theoretical frameworks in the rapid evolution of cardiac care, a thorough evaluation of existing theories is needed to ensure their applicability and relevance in improving patient care and education. One such theory is Adherence Theory for patients with chronic disease, originally developed by Kyngäs and refined by other researchers.^9,10^ This theory has been identified as a valid framework for understanding adherence to healthy behaviors and medication adherence in people with chronic diseases. This middle-range theory defines adherence as an active, intentional, and responsible process in which patients collaborate with healthcare professionals to maintain their health. It encompasses both medication adherence and lifestyle choices, such as maintaining a healthy diet, being physically active, not smoking, and consuming alcohol in moderation. Adherence Theory for patients with chronic disease has been validated as a suitable framework for evaluating treatment adherence in patients with CHD after PCI. Key explanatory factors include responsibility; cooperation; sense of normality; motivation; support from family, nurses, and physicians; and fear of complications.^9^

Recent studies indicate that adequate information about the disease and treatment options are critical to supporting patient adherence.^11^ On the basis of this evidence, the original Adherence Theory for patients with chronic disease has been expanded to include informational support. Informational support, as part of social support, is the provision of advice, counseling, and information to help people understand and cope with their problems.^12^ For people with CHD after PCI, informational support is essential to help them understand their condition, treatment options, and the lifestyle changes needed to manage their health.^13^ This support can improve medication adherence and promote healthier behaviors, ultimately improving patient outcomes.^14^

The results of the theory development suggest that informational support is a well-justified extension of the original adherence theory for patients with chronic disease. This added dimension of informational support provides a novel perspective for improving patient-centered care and counseling, thereby promoting adherence in people with lifelong conditions.^14^ However, it remains unclear whether the theoretical model evolves over time with longitudinal follow-up and whether it applies similarly across genders.

The objective of this study was to address the aforementioned gaps by examining and identifying associations within Adherence Theory for patients with chronic disease after PCI over the period of a long-term follow-up. It was hypothesized that Adherence Theory for patients with chronic disease would remain consistent over time and across genders in post-PCI patients.

Research questions are the following:

1. Is Adherence Theory for patients with chronic disease after PCI robust over a 6-year follow-up period (2013 vs 2019)?
2. Does Adherence Theory for patients with chronic disease after PCI differ between genders at baseline (2013) and long-term follow-up (2019)?

## Methods

### Study Design and Sample

A descriptive and exploratory study design was used in this study. A survey was conducted at 2 points: 4 months after PCI (2013) and 6 years later (2019). In 2013, after being informed about the study, 520 of the 572 eligible patients provided informed consent after being informed about the study. The sample size was sufficient to detect statistical significance with 80% power and a significance level of .05 for weak correlations (0.14). The number of observations and the incidence rate allowed for the detection of a 7% to 13% difference between the groups.

### Measurements

Data were collected using a postal questionnaire comprising the following instruments:

1. The Adherence of Patients with Chronic Disease Instrument (ACDI; see Table 1), which has been developed to assess patient adherence^15^ and has been tested and found to be valid among patients with CHD after PCI.^16^ The ACDI consists of 37 items. Responses are rated on a 5-point Likert scale ranging from 1 (strongly agree) to 5 (strongly disagree). A lower score indicates a stronger association between the variable and treatment adherence. The ACDI includes 3 negative mean sum variables as the reverse statements enhance the reliability of responses, verify consistency, and mitigate the priming effect by encouraging respondents to carefully read and evaluate each question individually.^13^

**TABLE 1.** Factors, Factor Loadings, and Cronbach *α*s Related to Mean Sum Variables of Adherence (N = 416)

Mean Sum Variables and Factor Loadings	Factor Loading	Cronbach *α*
Mean sum variables related to adherence to treatment		Total, 0.84
Adherence to medication	0.37–0.80	0.69
Item 1: Related to patient’s adherence to medication instructions		
Item 2: Related to patient’s medication changes		
Adherence to healthy lifestyle		0.53
Item 3: Related to patient’s smoking habits		
Item 4: Related to patient’s alcohol consumption		
Item 5: Related to patient’s physical activity		
Item 6: Related to patient’s diet		
Cooperation	0.37–0.87	0.71
Item 7: Related to patient’s secondary prevention follow-up treatment		
Item 8: Related to possibility for patient to discuss with physician		
Item 9: Related to possibility for patient to discuss with nurse		
Responsibility	0.40	0.41
Item 10: Related to patient’s own responsibility		
Item 11: Related to patient’s willingness to practice good self-care		
Support from next of kin	0.30–0.86	0.60
Item 16: Related to support from next of kin for patient’s self-care		
Item 25: Related to acceptance and support from next of kin		
Item 26: Related to how interested next of kin are in patient’s life		
Item 27: Related to how next of kin remind patient about treatment		
Item 28: Related to how next of kin motivate patient to self-care		
Sense of normality	0.26–0.84	0.88
Item 14: Related to patient’s refusal of treatment regimens		
Item 18: Related to patient’s inability to live a normal life		
Item 19: Related to patient’s willingness to stay at home because of illness		
Item 20: Related to how patient experiences self-care as a part of life		
Item 21: Related to how self-care limits patient’s independence		
Item 22: Related to how self-care limits patient’s daily routine		
Item 23: Related to how self-care causes dependence on next of kin		
Motivation	0.47	0.65
Item 13: Related to fatigue		
Item 15: Related to lack of motivation		
Results of care	0.40	0.40
Item 17: Related to the maintenance of health status		
Item 24: Related to well-being		
Support from nurses	0.62–0.90	0.60
Item 33: Related to nurse’s ability to make a complete plan for the patient’s care		
Item 34: Related to nurse’s complete interest in the patient		
Item 35: Related to nurse’s ability to motivate the patient		
Item 36: Related to nurse’s interaction skills		
Support from physicians	0.61–0.89	0.88
Item 29: Related to physician’s ability to make a complete plan for the patient’s care		
Item 30: Related to physician’s complete interest in the patient		
Item 31: Related to physician’s ability to motivate the patient		
Item 32: Related to physician’s interaction skills		
Fear of complications		0.88
Item 37: Related to patient’s fear of cardiac events		
Item 38: Related to patient’s fear of comorbidities		

Various aspects of adherence are assessed by the ACDI using 11 subscales: adherence to medication (2 items), adherence to a healthy lifestyle (4 items), responsibility (2 items), motivation (2 items; negative factor), cooperation (2 items), results of care (2 items), fear of complications (2 items; negative factor), sense of normality (7 items; negative factor), support from next of kin (5 items), support from nurses (4 items), and support from physicians (4 items).

High validity (both criterion and construct) and reliability (internal consistency) have been demonstrated for the ACDI in previous studies.^9,10,17^ In this study, an exploratory factor analysis with principal axis factoring and promax rotation was conducted to assess the construct validity of the ACDI. A factor solution with satisfactory statistical values was yielded by the exploratory factor analysis, as shown in Table 1. Missing data were replaced using the mean value for each item. The internal consistency of the mean sum variables was assessed using Cronbach *α* values, which ranged from 0.40 to 0.90, indicating a satisfactory to high degree of internal consistency. The total scale had a Cronbach *α* of 0.84, indicating high internal consistency.

2. The Social Support for People with Coronary Heart Disease instrument (Table 2) assesses the perceived social support in patients with CHD.^14^ The original Social Support for People with Coronary Heart Disease instrument consists of 14 items that assess social support in 3 categories: informational support (7 items), emotional support (4 items), and functional support (3 items). Responses are rated on a 5-point Likert scale, ranging from 5 (strongly disagree) to 1 (strongly agree). A lower score indicates stronger perceived support. The informational support dimension includes items such as advice about exercise after PCI, advice about risk factors, understanding personal risk factors, advice about managing chest pain, information about medications, and details about care and rehabilitation. The emotional support dimension includes perceived support from other cardiac patients, family, and friends, and the importance of the patient to the patient’s next of kin. The functional support dimension includes opportunities to ask questions, feelings of support and care, and cooperation with healthcare professionals.

**TABLE 2.** Factors, Factor Loadings, and Cronbach *α*s Related to Mean Sum Variables of Perceived Social Support (N = 416)

SSCHD Instrument	Factor Loading	Cronbach *α* (Total, 0.78)
Factor 1: Informational support	0.50–0.72	0.84
Information on the continuum of care and rehabilitation		
Information on medication		
Information on physical exercise after PCI		
Advice on own risk factors		
Information on how to act in the case of chest pain		
Information on CHD		
Knowledge of own risk factors		
Factor 2: emotional support	0.34–0.75	0.60
Support from friends		
Support from family		
Importance to next of kin		
Support from other patients		
Factor 3: Functional support	0.81–0.86	0.90
Healthcare professionals care about patient coping with CHD		
Opportunity to ask healthcare professionals about issues of concern		
Support from healthcare professionals		

Abbreviations: CHD, coronary heart disease; PCI, percutaneous coronary intervention; SSCHD, Social Support for People with Coronary Heart Disease instrument.

Construct validity was confirmed through exploratory factor analysis, which used principal axis factoring and promax rotation, with missing values replaced by item means. Cronbach *α* of 0.84 indicated acceptable internal consistency for the informational support dimension. In addition, 3 nurses with extensive experience in cardiovascular nursing and 15 patients with CHD who had previously undergone PCI were used to assess the face validity of the questionnaire. Only the informational support scale was used in this study because emotional and functional support items are covered by the ACDI.

### Ethical Considerations

The ethical review board of the University Hospital of Kuopio (ref. 74//2012, anonymized), and each research center separately approved the study. Conducted in accordance with the ethical principles of the Finnish Advisory Board on Research Integrity (2021) and the Declaration of Helsinki, patients gave informed consent before being discharged from hospital. They received verbal and written information about the study from a registered nurse and were informed that participation was voluntary and that they could withdraw at any time. They were given the contact details of the researchers for any further questions and were assured of confidentiality. Data were collected by postal questionnaire 4 months and 6 years after PCI, anonymized using codes, and analyzed confidentially.

### Data Analysis

Analysis of Moment Structures (AMOS) version 27 was used to analyze the data. The hypotheses were tested using structural equation modeling to better understand the relationship between the factors in Adherence Theory for chronic disease patients, comparing the periods after PCI (2013 vs 2019) and also examining gender differences during these periods.^14^ In this study, a χ^2^ test and its derivatives were used to assess the compatibility between the established theoretical models and the observed correlation matrix.^16^ In terms of standardized regression weights, a weak effect between factors was indicated by values less than 0.10; a moderate effect, by values around 0.30; and a strong effect, by values greater than 0.5.^14,17^

## Results

### Sample Characteristic

Four months after PCI at 2 Finnish university hospitals and 3 central hospitals, 416 patients (81% response rate) completed a postal questionnaire. All eligible patients were invited to participate in the study using convenience sampling. The initial inclusion criteria were that participants should be between 18 and 75 years old, having adequate cognitive function, and having no memory impairment. Patients were recruited into the study by the attending nurse, who determined their eligibility for participation based on a comprehensive assessment that included observation and review of the patient’s medical records. Of the original participants, 352 (84.6%) agreed to remain in the study for follow-up questionnaires. Six years later, 167 patients (40.1% of the original participants) completed the second set of questionnaires.

The mean age of respondents at baseline (2013) was 63.3 ± 7.5 years. Most respondents were male (n = 312, 75.5%) and reported being married or in a close personal relationship (n = 320, 77.1%). A significant proportion of respondents (n = 251, 60.6%) were retired and had been living with CHD for a mean of 4.7 ± 7.4 years. Before the index PCI (2013), 45 patients (10.8%) had undergone PCI, and 21 patients (5.0%) had undergone coronary artery bypass grafting. At the 6-year follow-up (2019), the participants were naturally older with a mean age of 68.5 ± 7.0 years. The number of men was still higher than the number of women, but the proportion was almost the same as at baseline (76.9% male). At the follow-up, there was a slight improvement in the health behaviors of the participants in terms of increased physical activity, increased vegetable consumption, and decreased alcohol consumption and smoking. In addition, systolic blood pressure, total cholesterol, and low-density lipoprotein cholesterol levels were more likely to be at target levels at follow-up compared with baseline results in 2013. The data indicate that, between PCI at baseline and the 6-year follow-up, 3 patients (1.9%) underwent repeat PCI, and 1 patient (0.6%) underwent coronary artery bypass grafting (Table 3). No information was available about the patients who did not participate in the 2019 follow-up study.

**TABLE 3.** Disease-Specific, Sociodemographic, and Health Behavioral Characteristics of Patients 4 Months and 6 Years After Percutaneous Coronary Intervention

Factors	Baseline 2014 (n = 154)	Follow-up 2019 (n = 169)
	n (%), mean ± SD	n (%), mean ± SD
Sociodemographic factors		
Gender, male	312 (75.5)	130 (76.9)
Age, y	63.3 ± 7.5	68.5 ± 7.01
Married/close relationship	320 (77.1)	130 (77.4)
Retired	251 (60.6)	131 (78.0)
Health behavioral factors		
Physical activity ≥ 120 min/wk	1185 (44.5)	80 (48.2)
Vegetable consumption, dL/d	2.3 ± 1.3	2.5 ± 1.2
Alcohol consumption, ≤2 portions	67 (49.3)	99 (61.9)
Nonsmoking	351 (84.6)	154 (91.1)
Disease-specific factors		
Systolic blood pressure		
≤139 mm Hg	279 (67.1)	120 (73.6)
Total cholesterol		
≤4.5 mmo1/L	218 (79.3)	81 (84.1)
LDL cholesterol		
≤1.8 mmol/L	96 (37.9)	39 (44.8)
Several PCI (in addition to index)	45 (10.8)^a^	3 (1.9)^b^
CABG	21 (5.0)^a^	1 (0.6)^b^
Duration of CHD, y	4.67 ± 7.4	10.2 ± 8.0

The values denote mean ± SD or n (%).

Abbreviations: AMI, acute myocardial infarction; CABG, coronary artery bypass grafting; CHD, coronary heart disease; LDL, low-density lipoprotein; PCI, percutaneous coronary intervention.

a Before index PCI.

b Between 2013 and 2019.

### Structural Equation Modeling

#### The Baseline of the Study

**FIGURE 1.**
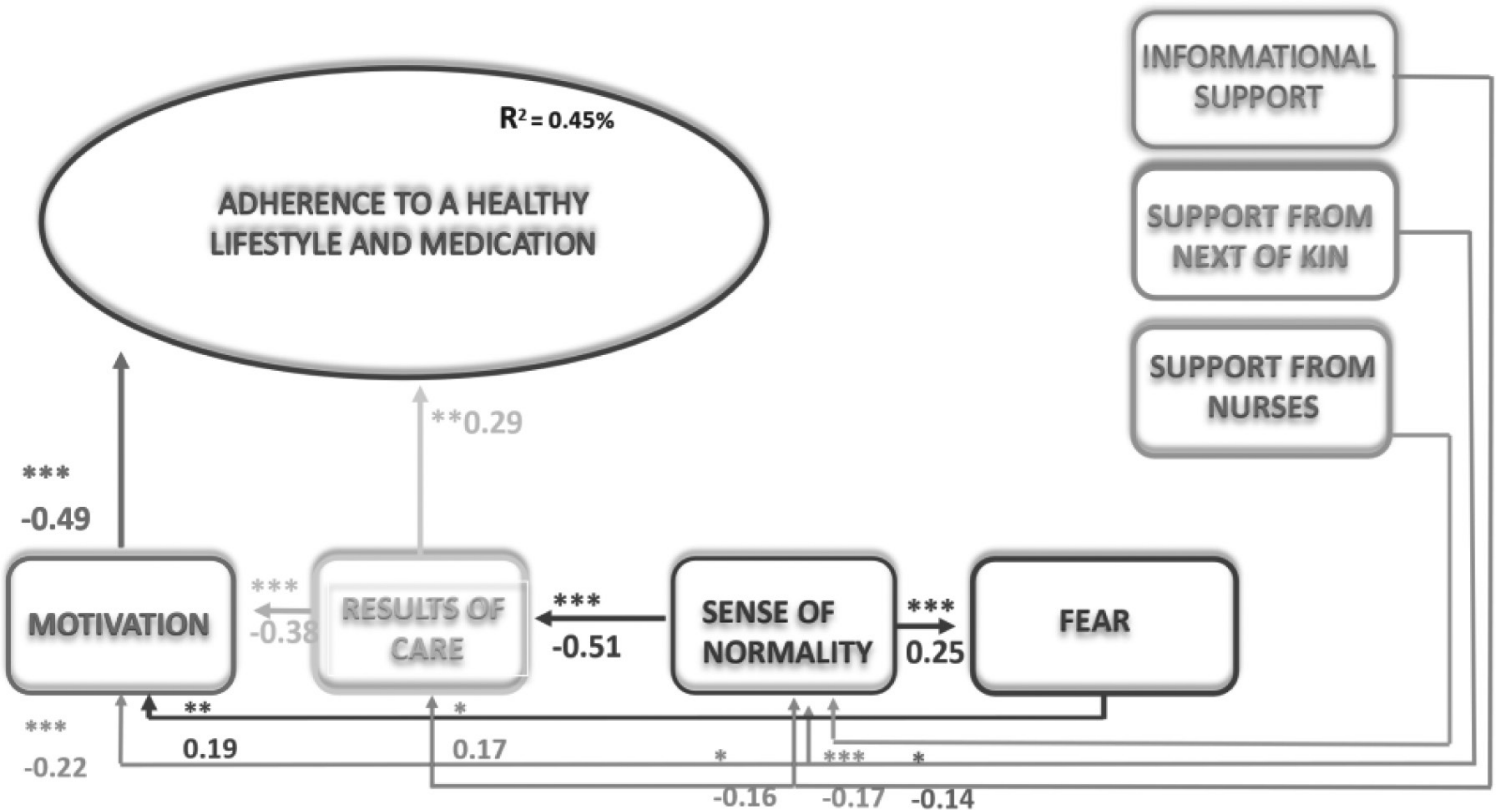
Extended theory of adherence to treatment of post–percutaneous intervention patients (Kähkönen et al, 2024). *P < .05, **P < .01, ***P < .001.

The baseline (2013) results have been reported previously^14^ (Figure 1), confirming the following factor associations: direct associations between the factors motivation and adherence to treatment, with a β coefficient of −0.48 (*P* < .001), and between results of care and adherence to treatment, with a β coefficient of 0.29 (*P* = .01). Results of care (β = −0.38, *P* < .001), fear of complications (β = 0.18, *P* = .01), and support from next of kin (β = 0.22, *P* = .003) were associated with motivation and thus indirectly through the mediating effect of motivation. Furthermore, indirect associations were confirmed between sense of normality and results of care (β = −0.51, *P* < .001), informational support (β = −0.16, *P* = .02), support from next of kin (β = 0.17, *P* = .02), and support from nurses (β = −0.14, *P* = .02) with sense of normality, and sense of normality with fear of complications (β = 0.25, *P* < .001). This model explained 45% of the variance in the factors associated with adherence.

#### The First Phase of the Study

**FIGURE 2.**
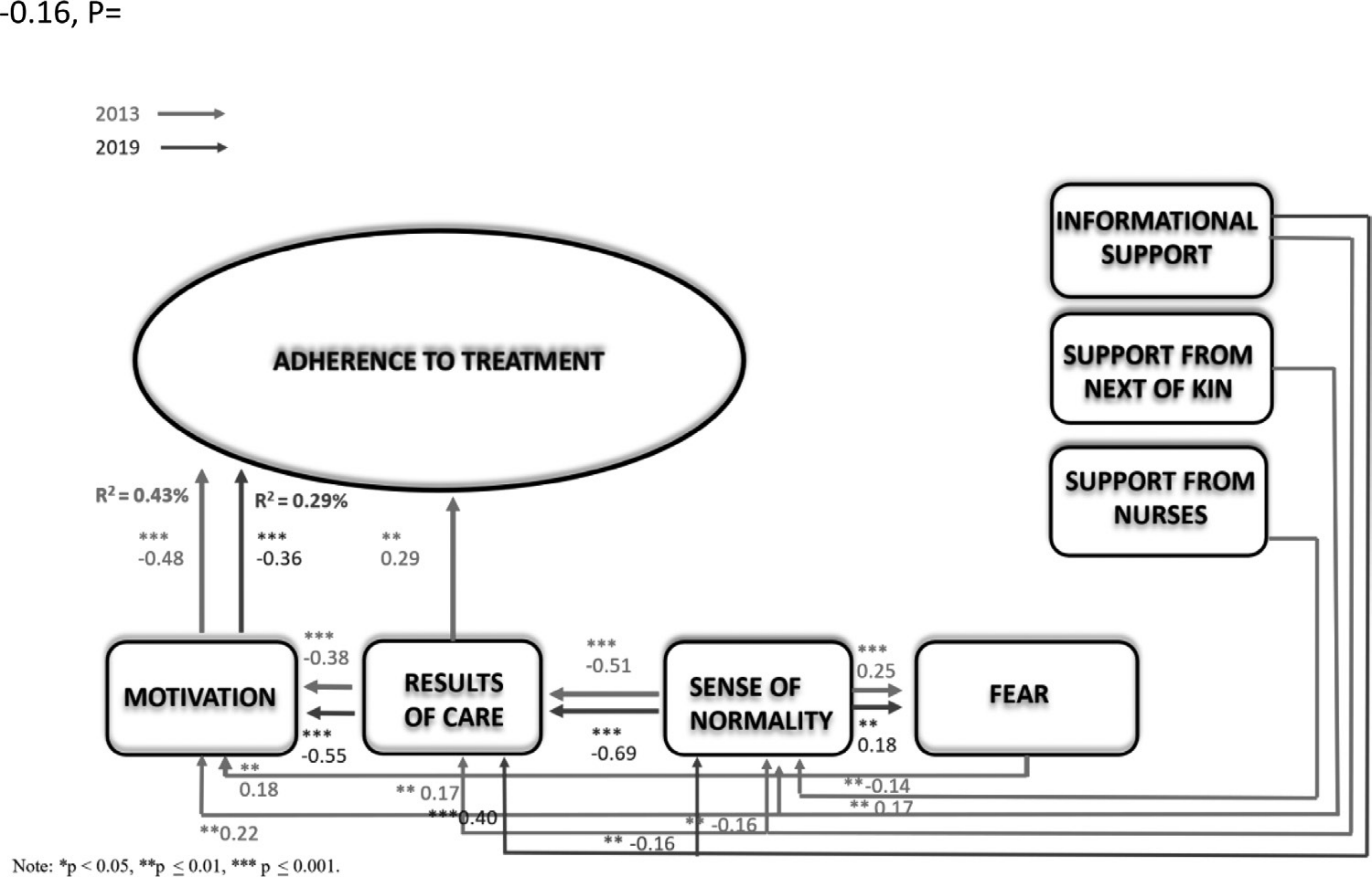
The extended theory of adherence—a comparison between 2 points in time: 2013 and 2019.

In this study, Adherence Theory for patients with chronic diseases was tested in the first phase using follow-up data from 2019. The generated model was compared with the baseline (2013) with the same assumptions (n = 167; Figure 2, Table 4). Despite the differences in the 2013 and 2019 models, the results showed no statistically significant difference (∆χ^2^/11 *df* = 18.36, *P* = .07). In 2019, motivation was still directly related to adherence (β = −0.36, *P* ≤ .001). In addition, informational support continued to have an indirect effect on adherence through a sense of normality (β = −0.16, *P* = .003), results of care (β = 0.40, *P* < .001), and motivation (β = −0.55, *P* ≤ .001).

**TABLE 4.** Testing Adherence Theory for Patients With Chronic Diseases Among Post–Percutaneous Intervention Patients Between Genders at the Start of the Study in 2013 (Baseline, N = 416)

Hypothesized Relationships	Female 2013 Standardized Estimates (*t*), *P*	Male 2013 Standardized Estimates (*t*), *P*	Group Differences ∆χ^2^/1 *df*
Whole model			21.96, *P* = .02
Motivation and adherence to treatment	−0.57 (−2.18), *P* = .01	−0.52 (−4.18), *P* ≤ .001	2.55, *P* = .11
Informational support and sense of normality	−0.22 (−1.49), *P* = .14	−0.18 (−2.44), *P* = .02	0.55, *P* = .46
Support from next of kin and sense of normality	0.02 (0.16), *P* = .87	0.23 (2.61), *P* = .01	1.13, *P* = .26
Sense of normality and results of care	−0.06 (−0.49), *P* = .62	−0.64 (−5.54), *P* ≤ .001	8.88, *P* = .003
Informational support and results of care	0.03 (0.23), *P* = .82	0.22 (2.44), *P* = .02	0.40, *P* = .53
Results of care and motivation	−0.17 (−1.23), *P* = .22	−0.46 (−4.00), *P* ≤ .001	1.66, *P* = .20
Sense of normality and fear of complications	0.20 (2.29), *P* = .01	0.27 (3.93), *P* ≤ .001	0.15, *P* = .70
Support from next of kin and motivation	0.31 (1.85), *P* = .22	0.15 (0.08), *P* = .08	0.67, *P* = .20
Support from nurses and sense of normality	−010 (−0.85), *P* = .40	−0.13 (−1.89), *P* = .06	0.1, *P* = .73

#### The Second Phase of the Study

**FIGURE 3.**
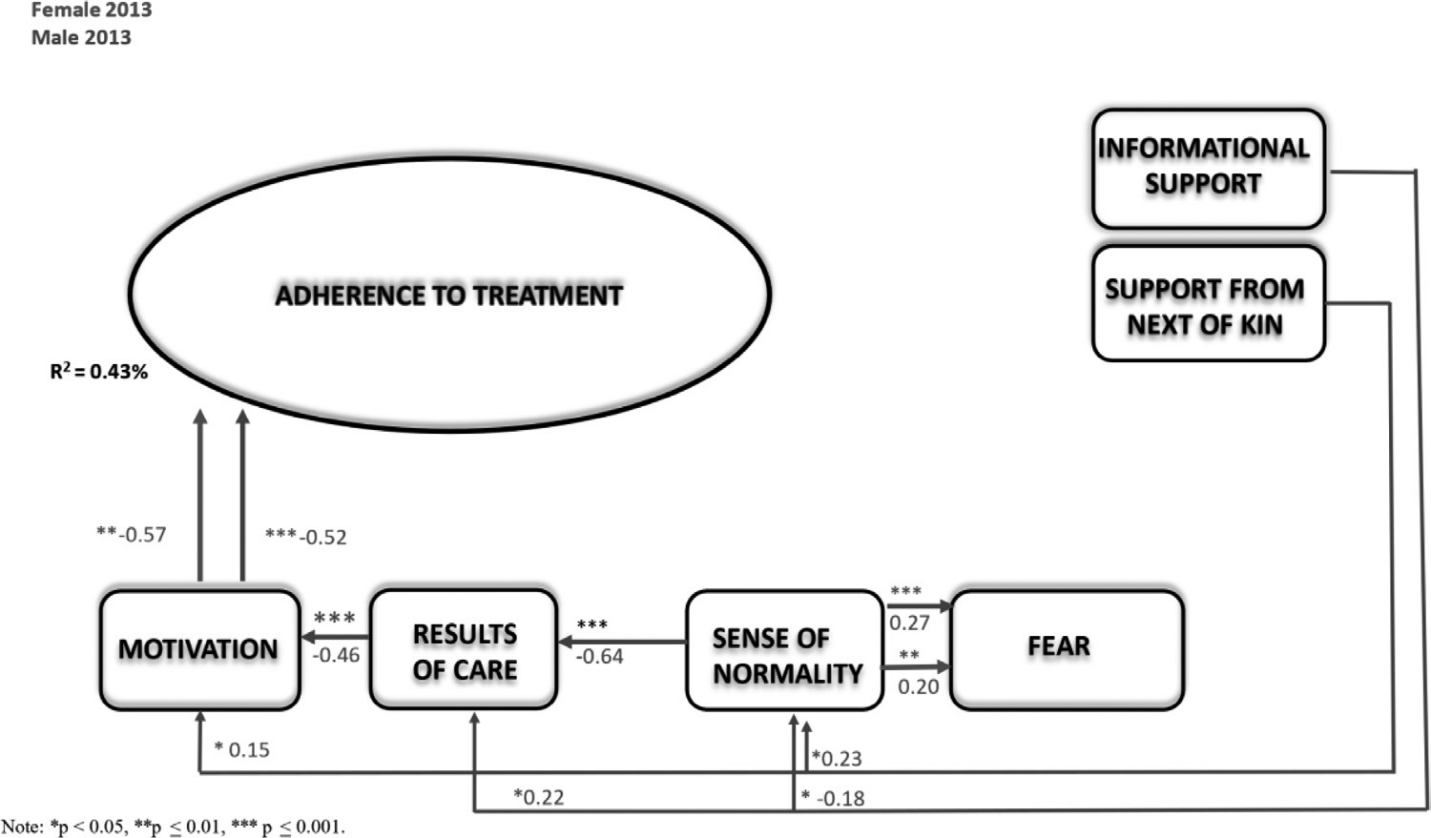
The extended theory of adherence comparison between genders at the start of the study in 2013.

**FIGURE 4.**
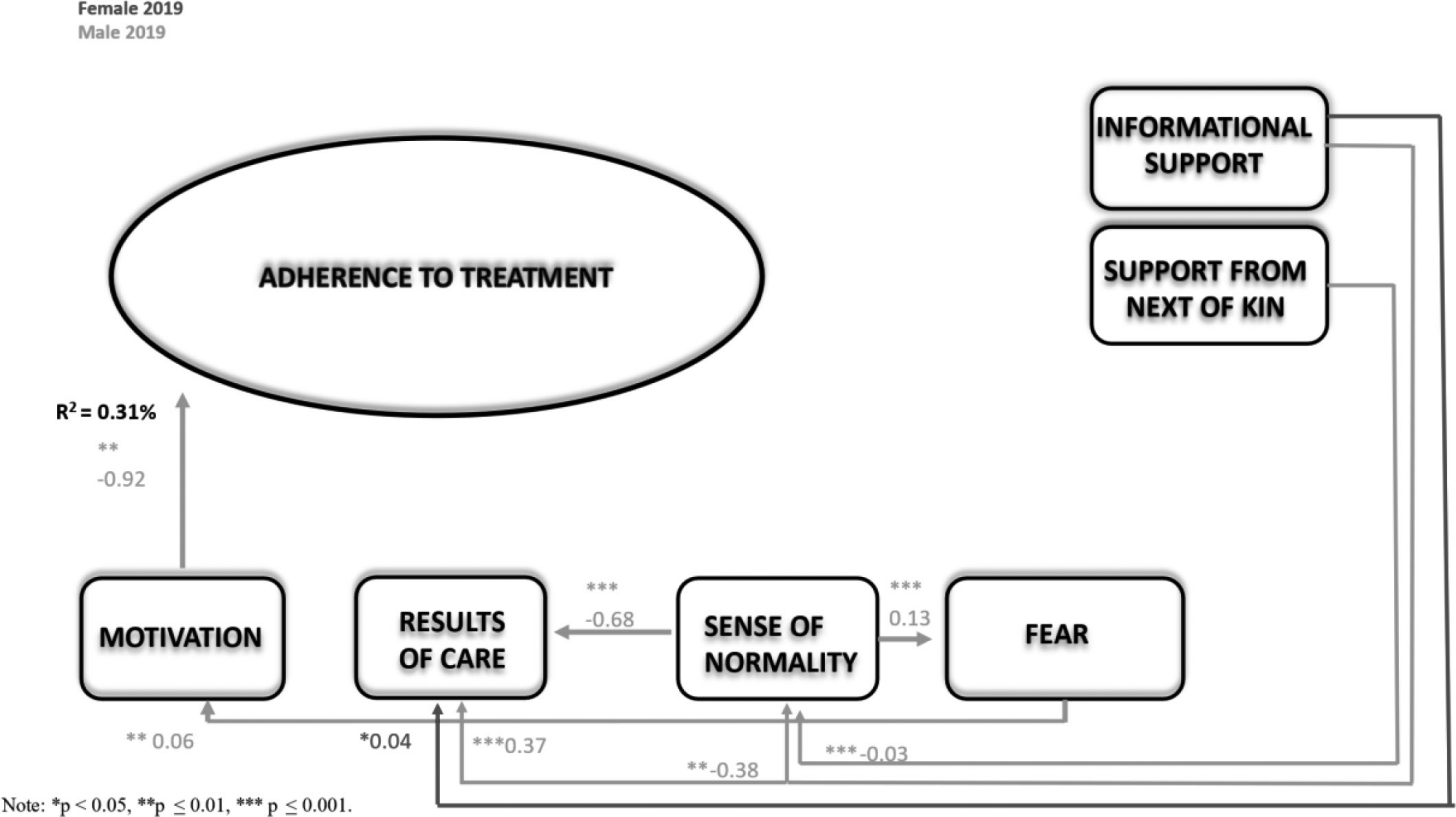
The extended theory of adherence comparison between genders at the 6-year follow-up in 2019.

Then, in the second phase of this study, the authors examined Adherence Theory for patients with chronic disease after PCI in the structure in 2013 (Figure 3, Table 5) and 2019 (Figure 4, Table 6) between genders and found a statistically significant difference (∆χ^2^/10 *df* = 21.96, *P* = .02; Table 3). In the female model in 2013, there was only an association between motivation and adherence to treatment (β = −0.57, *P* = .01). In the male model, there was also a direct association between motivation and adherence to treatment (β = −0.52, *P* < .001). In addition, an indirect association was found in the male model between informational support and sense of normality (β = −0.18, *P* = .02), support from next of kin and sense of normality (β = 0.23, *P* = .01), sense of normality and results of care (β = −0.64, *P* < .001), informational support and results of care (β = 0.22, *P* = .02), and results of care and motivation (β = −0.46, *P* < .001). Examining how each factor differed by gender in 2013 revealed statistically significant differences between sense of normality and results of care (∆χ^2^/1 *df* = 8.88, *P* = .003). The model explained 43% of the variance in the factors associated with adherence to treatment.

**TABLE 5.** Testing the Extended Theory of Adherence Among Post–Percutaneous Intervention Patients Between Genders: 6-Year Follow-up in 2019 (N = 167)

Hypothesized Relationships	Female 2019 Standardized Estimates (*t*), *P*	Male 2019 Standardized Estimates (*t*), *P*	Group Differences ∆χ^2^/1 *df*
Whole model			38, 1/10 *P* < .001
Motivation and adherence to treatment	0.06 (0.94), *P* = .34	−0.92 (−2.59), *P* = .01	7.18, *P* = .01
Sense of normality and results of care	−0.25 (−1.65), *P* = .1	−0.68 (−3.60), *P* < .001	0.47, *P* = .49
Sense of normality and fear of complications	0.18 (1.01), *P* = .32	0.13 (−3.62), *P* < .001	0.50, *P* = .49
Fear of complications and motivation	0.14 (0.29), *P* = .77	0.06 (−2.63), *P* = .01	0.83, *P* = .77
Support from next of kin and sense of normality	0.54 (0.79), *P* = .79	−0.03 (3.44), *P* < .001	4.35, *P* = .04
Results of care and adherence to treatment	−0.12 (−0.63), *P* = .53	0.07 (0.21), *P* = .83	0.07, *P* = .79
Results of care and motivation	0.13 (0.28), *P* = .32	−0.75 (−5.03), *P* = .98	10.1, *P* = .002
Support from nurses and sense of normality	0.18 (0.93), *P* = .36	0.15 (−0.23), *P* = .82	0.1, *P* = .91
Support from next of kin and motivation	0.19 (0.20), *P* = .84	0.30 (1.50), *P* = .14	−0.11, *P* = 1.0
Informational support and sense of normality	−0.21 (−0.92), *P* = .36	−0.38 (−2.62), *P* = .01	0.08, *P* = .77
Informational support and results of care	0.32 (2.17), *P* = .03	0.37 (3.50), *P* < .001	0.71, *P* = .40

**TABLE 6.** Testing Adherence Theory for Patients With Chronic Diseases Among Post–Percutaneous Intervention Patients at 2 Time Points: 2013 (Baseline, n = 416) and 2019 (Follow-up, n = 167)

Hypothesized Relationships	2013 Standardized Estimates (*t*), *P*	2019Standardized Estimates (*t*), *P*	Group Differences ∆χ^2^/1 *df*
Whole model			15.8/9 *df* *P* = .07
Motivation and adherence to treatment	−0.48 (−4.07), *P* < .001	−0.36 (−1.78), *P* = .05	0.21, *P* = .65
Results of care and adherence to treatment	0.29 (2.51) *P* = .01	0.25 (1.25), *P* = .18	0.07, *P* = .79
Results of care and motivation	−0.38 (−4.81), *P* < .001	−0.55 (−4.12), *P* < .001	1.28, *P* = .26
Fear of complications and motivation	0.18 (2.82), *P* = .01	0.14 (1.63), *P* = .10	0.43, *P* = .51
Support from next of kin and motivation	0.22 (2.94), *P* = .003	0.29 (1.56), *P* = .11	0.69, *P* = .41
Sense of normality and results of care	−0.51 (−5.54), *P* < .001	−0.69 (−4.22), *P* < .001	2.4, *P* = .12
Informational support and results of care	0.17 (2.86), *P* = .004	0.40 (3.38), *P* < .001	0.38, *P* = .54
Informational support and sense of normality	−0.16 (−2.35), *P* = .02	−0.39 (−2.99), *P* = .003	0.13, *P* = .72
Support from next of kin and sense of normality	0.17 (2.41), *P* = .02	0.004 (0.05), *P* = .96	1.27, *P* = .26
Support from nurses and sense of normality	−0.14 (−2.29), *P* = .02	0.16 (1.49), *P* = .14	7.35, *P* = .01
Sense of normality and fear of complications	0.25 (4.54), *P* < .001	0.18 (2.38), *P* = .02	0.03, *P* = .87

The results showed that, in 2019 (Figure 4, Table 5), Adherence Theory for patients with chronic disease after PCI was statistically significantly different between women and men (∆χ^2^/10 *df* = 38.1, *P* < .001). None of the factors in the female model explained adherence to treatment. The only statistically significant association was found for informational support for results of care (β = 0.32, *P* = .03). In contrast, in the male model, a statistically significant direct association was found between motivation and adherence to treatment (β *=* −0.92, *P* = .01), and an indirect association was found between sense of normality and results of care (β = −0.68, *P* < .001), sense of normality and fear of complications (β = 0.13, *P* > .001), fear of complications and motivation (β = 0.06, *P* = .01), support from next of kin and sense of normality (β = −0.03, *P* < .001), informational support and sense of normality (β = −0.38, *P* = .01), and informational support and results of care (β = 0.37, *P* < .001). When examining how each factor differed by gender in 2019, statistically significant differences were found between the following: motivation and adherence to treatment (∆χ^2^/1 *df* = 7.18, *P* = .01), results of care and motivation (∆χ^2^/1 *df* = 10.1, *P* = .002), and support from next of kin and sense of normality (∆χ^2^/1 *df* = 4.35, *P* = .04).

## Discussion

Nursing theories often fail to evolve alongside advancements in nursing practice, which reduces their relevance to contemporary realities. In this study, the authors investigated the robustness of Adherence Theory for patients with chronic disease in the long-term (6 years) follow-up after PCI in patients with CHD. A descriptive and exploratory design was used to collect baseline data in 2013 and follow-up data in 2019 from 5 Finnish hospitals. Structural equation modeling was used to test the model, focusing on patient adherence and social support.

Patient adherence to treatment after PCI is essential for maintaining favorable outcomes and preventing disease progression.^18^ Adherence Theory for chronic disease patients has been identified as an appropriate theoretical framework for counseling patients after PCI.^14^ Given the importance of assessing the long-term relevance of this theory, in this study, the authors evaluated Adherence Theory for patients with chronic disease after PCI using structural equation modeling, a statistical method designed to test theoretical models. Structural equation modeling is consistent with the current trends in nursing research that emphasize the use of advanced statistical techniques to evaluate and validate nursing theories.^19^

A notable finding of this study was the absence of statistically significant differences in Adherence Theory construct for patients with chronic disease after PCI at 2 different time points (2013 vs 2019) during long-term follow-up. This suggests that the theory is robust in terms of treatment adherence over time. However, examining the theory’s constructs based on gender revealed significant differences at baseline and at the long-term follow-up stage. In this respect, the theory is not robust. This finding is important because it supports the need to consider gender differences when designing patient education and nursing interventions.

Motivation was directly related to adherence for both genders in 2013 but only for men in the long-term follow-up in 2019. Previous studies have also shown a clear association between motivation and adherence.^20–22^ Therefore, motivating patients with chronic conditions such as CHD requires special attention. A motivational approach is a client-centered strategy based on needs and values that increases motivation and changes harmful behaviors. Motivational communication has several important advantages. It is patient centered and respectful of self-determination, allowing patients to define their own goals and how to achieve them.^22^

The results of this study indicate substantial differences in adherence profiles between male and female patients at baseline and over time. Adherence Theory for patients with chronic diseases does not sufficiently explain the long-term adherence model observed in women after PCI. At baseline, only motivation was associated with adherence. This finding is notable. Adherence is especially crucial for female patients because studies have demonstrated poorer long-term outcomes, higher rates of comorbidities, major adverse cardiac events, and bleeding episodes in this group. This issue is more pronounced in female patients than in male patients of the same age undergoing PCI,^23^ despite that female patients tend to have a lower risk profile.^24^

Previous research suggests that women, particularly those with lower levels of education, exhibit poorer lifestyle adherence than men.^25^ In addition, they receive less comprehensive counseling for managing lifestyle risk factors. Although women are more likely to adhere to a healthier diet, they are less likely to increase their physical activity levels.^26,27^ The CHD prognosis after PCI highlights the need for targeted interventions that take gender differences into account.^28^

Examining the factors associated with adherence between genders revealed the importance of informational support. Although the theoretical model for women did not show an association between informational support and adherence, informational support was associated with perceived positive results of care during the long-term follow-up. In comparison, the male theoretical model revealed that informational support was indirectly associated with a sense of normality, indicating that one can live a normal life with the disease. Furthermore, a sense of normality through fear of complications was associated with motivation, which consequently led to adherence. Previous studies have shown that female and male patients have different knowledge expectations, with women having higher expectations than men.^29^ In addition, gender differences in postoperative stress reactions and anxiety underscore the importance of providing women with appropriate patient education to mitigate these disparities.^30^ Moreover, patients require information and knowledge to motivate them to take their medications and enhance treatment adherence.^31^

The results of this study showed that support from next of kin explained the greater positive effects observed for men than for women. Previous research has shown that married men are less likely to engage in unhealthy, high-risk behaviors and tend to have better health outcomes.^32^ On the other hand, the social environment, cultural influences, and social norms affecting women must also be considered, because these factors may make it more difficult for women to prioritize changing their health behaviors.^33^

The results underscore the importance of critically reassessing nursing theories to ensure their relevance. Nursing theories have historically adapted to changes in healthcare and a deeper understanding of the patient’s experience. Theories continue to evolve as nurses and theorists engage with new evidence, technologies, and societal challenges, ensuring the continued relevance and evolution of nursing knowledge and practice.^6,34^ To remain dynamic and useful, nursing needs to embrace new ideas and evidence-based approaches. The use of nursing theory in research is often inadequate, and outcome studies lack theoretical context. A major problem is the artificial separation of theory and practice, which reduces the visibility and impact of nursing theories.^8^

### Limitations of the Work

First, the use of self-reported data collection methods is susceptible to social desirability bias, whereby patients may provide answers that they perceive as favorable rather than accurately reflecting their true beliefs or behaviors. Second, there is a potential for bias in the recruitment process for the 2013 baseline study. Given that patients are typically discharged 24 hours after PCI, it is possible that some eligible patients may have been missed because of the rapid turnover. Third, it is important to acknowledge the potential for respondent bias, which is a significant limitation of this study. At baseline, participants were asked for consent to be contacted for further research at follow-up. Of the 416 patients who participated in the study at baseline in 2013, 352 (84.6%) agreed to be contacted for follow-up. However, the final response rate at 6 years was 40.1% (n = 167), leaving a relatively low number of female participants, although the proportion was similar between the baseline and follow-up data. In addition, it is noted that patients who are effectively adherent to their treatment are more likely to respond to questionnaires. Fourth, according to the protocol, the results were analyzed at the group level. Finally, the original data were collected in 2013, with a follow-up 6 years later. Although treatment practices have not changed significantly, the passage of time should still be considered, because it may limit the generalizability of the findings. Given these limitations, the results should be interpreted with caution.

## Conclusion

The results underscore the importance of critically reassessing nursing theories to ensure their relevance. This research-based knowledge can then be used to develop nursing interventions that promote person-centered care. Adherence Theory for chronic disease patients is robust over time, but gender differences need to be recognized in nursing practice. Significant gender differences in adherence and health outcomes highlight the need for targeted, gender-specific interventions. Therefore, nursing practice should consider these factors to effectively improve patient outcomes.
